# Targeted Modification of the Foot-And-Mouth Disease Virus Genome for Quick Cell Culture Adaptation

**DOI:** 10.3390/vaccines8040583

**Published:** 2020-10-03

**Authors:** Veronika Dill, Aline Zimmer, Martin Beer, Michael Eschbaumer

**Affiliations:** 1 Institute of Diagnostic Virology, Friedrich-Loeffler-Institut, Federal Research Institute for Animal Health, 17493 Greifswald-Insel Riems, Germany; veronika.dill@gmx.de (V.D.); martin.beer@fli.de (M.B.); 2 Merck KGaA, Merck Life Sciences, Upstream R&D, 64293 Darmstadt, Germany; aline.zimmer@merckgroup.com

**Keywords:** foot-and-mouth disease virus, serotype Asia-1, BHK suspension cells, mutagenesis, particle stability, neutralizing antibody response, recombinant virus, vaccine production

## Abstract

Foot-and-mouth disease virus (FMDV) causes the highly contagious foot-and-mouth disease, which is characterized by the appearance of vesicles in and around the mouth and feet of cloven-hoofed animals. BHK-21 cells are the cell line of choice for the propagation of FMDV for vaccine production worldwide but vary in their susceptibility for different FMDV strains. Previous studies showed that the FMDV resistance of a certain BHK cell line can be overcome by using a closely related but permissive cell line for the pre-adaptation of the virus, but the adapted strains were found to harbor several capsid mutations. In this study, these adaptive mutations were introduced into the original Asia-1 Shamir isolate individually or in combination to create a panel of 17 Asia-1 mutants by reverse genetics and examine the effects of the mutations on receptor usage, viral growth, immunogenicity and stability. A single amino acid exchange from glutamic acid to lysine at position 202 in VP1 turned out to be of major importance for productive infection of the suspension cell line BHK-2P. In consequence, two traditionally passage-derived strains and two recombinant viruses with a minimum set of mutations were tested in vivo. While the passaged-derived viruses showed a reduced particle stability, the genetically modified viruses were more stable but did not confer a protective immune response against the original virus isolate.

## 1. Introduction

Foot-and-mouth disease (FMD) of cloven-hoofed animals is probably the most contagious disease known. FMD is endemic in many countries of Africa, Asia and the Middle East, where it causes a reduction in meat and milk production and is an obstacle in the international trade of animals and their products [1]. Its severe consequences threaten the livelihood of humans in enzootic areas who depend on their livestock [2]. 

FMD is caused by the foot-and-mouth disease virus (FMDV), which comprises seven distinct serotypes: A, O, C, Asia-1 and Southern African Territories 1-3. The significant genetic variability within and between serotypes leads to a lack of cross-protection between serotypes [3,4]. As with other positive-sense RNA viruses, the error-prone RNA-dependent RNA polymerase of FMDV is responsible for high mutation rates during virus replication [5]. 

The primary control measure for FMD is repeated prophylactic vaccination. Antigens for chemically inactivated whole-virus vaccines are industrially produced in baby hamster kidney 21 (BHK-21) cells [6]. For industrial-scale yields, they are adapted to grow in suspension and are cultured in large fermenters [7,8]. However, BHK-21 cells vary in their susceptibility for different FMDV strains and can become refractory to infection with repeated subculturing [9,10,11]. Serotype Asia-1 viruses are the most difficult to grow in BHK-21 cells and the adaptation of the viruses to these cells is linked to extensive amino acid exchanges in the viral capsid [9,10,12] that can compromise their utility for vaccine use [12]. 

A panel of 17 Asia-1 mutant viruses was constructed by reverse genetics and screened for replication on adherent and suspension cells with different receptor repertoires. To examine whether Asia-1 viruses adapted to suspension culture can still confer a protective immune response to the “wild type” virus isolate, two passage-derived strains and two recombinant viruses were grown in BHK-2P suspension cells, formulated into adjuvanted vaccines and injected into guinea pigs. The passage-derived strains Asia-#8 and Asia-#9 [12] failed to evoke a detectable immune response due to reduced stability of the virus particles. The recombinant viruses Asia-Mut 9-4 and Asia-Mut 9-7 induced antibodies that neutralized the homologous viruses in vitro, but did not neutralize the Asia-1 Shamir “wild type” isolate. 

## 2. Materials and Methods 

### 2.1. Cells and viruses 

All cell lines used for this study (see Table 1) were maintained as described previously [12]. The Asia-1 Shamir virus isolate was selected from archival stocks of the Friedrich-Loeffler-Institut in Greifswald, Germany (FLI). Its derivatives Asia-#8 and Asia-#9 were obtained by passaging, as described in [12]. 

### 2.2. Construction of Recombinant FMDV 

The full-length genome of Asia-1 Shamir was commercially synthesized (GeneArt, Thermo Fisher Scientific, Regensburg, Germany) based on the sequence deposited in GenBank (accession no. JF739177.1) and was delivered in two parts (“Asia-5p”, containing the genetic sequence from the 5’ end of the genome to nucleotide 429 of the 3A coding region, and “Asia-3p” from nucleotide 430 to the poly-A-tail). The cDNA of “Asia-5p” was digested with *Spe*I and *Xma*I, while the cDNA of “Asia-3p” was digested with *Xma*I and *Hpa*I and both parts were ligated into the pT7S3 backbone [13] previously digested with *Spe*I and *Hpa*I. The resultant plasmid was named pT7S3_Asia-1. All restriction enzymes were obtained from New England BioLabs GmbH, Frankfurt am Main, Germany (NEB). A naturally occurring *Hpa*I restriction site in the 2C coding region of Asia-1 Shamir was silently mutated by changing the codon in question from GTT to GTG using primers FMD-5143-F [14] and Asia 2C_GTT-GTG-Rev (5′ GGT TAT GTC CAA TTT GTT GTT CAC TTT GTA CTC GTC CTT GGC GC 3′). 

Point mutations were introduced in the VP1 and VP3 coding regions by site-directed mutagenesis and restriction-free cloning [15] using Phusion High-Fidelity DNA Polymerase (Thermo Fisher Scientific) and the following primers: Asia#8_VP1_Q108R-Fw (5′ CAA CCC AAC TGC CTA TCG GAA GCA ACC CAT CAC CCG CCT GG 3′), Asia#8_VP1_Q110R-Fw (5′ CAA CCC AAC TGC CTA TCA GAA GCG ACC CAT CAC CCG CCT GG 3′), Asia#8_Q108R_Q110R-Fw (5′ CAA CCC AAC TGC CTA TCG GAA GCG ACC CAT CAC CCG CCT GG 3′), Asia#9_VP3_E59K-Fw (5′ CCA ACT TTC CTC CGC TTT GGA AAA GTA CCA TTT GTG AAG AC 3′), Asia#9_VP1_T83A-Rev (5′ GCA CCC AGG TGA CCG GGC CTG CGT GGA CAA GCG CGA CCT CCA G 3′), Asia#9_VP1_Q110K-Fw (5′ CCC AAC TGC CTA TCA GAA GAA ACC CAT CAC CCG CCT GGC AC 3′), Asia#9_VP1_E202K-Rev (5′ CTT CTC AGG TGC AAT GAT CTT CTG TTT GCG GCG GTC CTG AG 3′) as well as the previously published primers FMD-4303-R, FMD-3223-R and FMD-3161-F [14]. The mutagenesis reactions were used for transformation of competent 10-beta *E. coli* cells (C3019, NEB). Positive colonies were confirmed by sequencing [14], then expanded and plasmid DNA was extracted using Plasmid Midi kits (Qiagen, Hilden, Germany). The mutated plasmids were linearized with *Hpa*I and in-vitro transcribed with the Standard RNA Synthesis Kit (E2040, NEB). The resulting cRNAs were purified using TRIzol LS Reagent (Life Technologies) in combination with the RNeasy Mini Kit (Qiagen, Hilden, Germany). The purified RNA was introduced into adherent BHK-21 cells with Lipofectamine 3000 (Thermo Fisher Scientific). The rescued viruses were passaged two times to generate a viral master seed. The introduction of the desired mutations was confirmed by sequencing [14].

### 2.3. Infectivity Assay 

Infectivity assays were performed in adherent BHK-21, Chinese hamster ovary K1 (CHO-K1) and CHO677 cells, as well as BHK-2P, BHK-InVitrus (#8) and production BHK (#9) suspension cells. Similar to the procedure described by Jackson et al. [16], cells were incubated with virus for 1 h at 37 °C. Virus that remained in the supernatant was inactivated by rinsing the cells with citric acid buffer at pH 5.2, before the cells were incubated for another 24 h at 37 °C. Viral RNA was extracted from the cultures using the NucleoMag VET Kit (Macherey-Nagel, Düren, Germany) on a KingFisher magnetic-particle-based extraction platform (Thermo Fisher Scientific). To screen the cultures for FMDV RNA, a real-time RT-PCR was performed using the AgPath-ID One-Step RT-PCR Kit (Applied Biosystems, Foster City, CA, USA) with the primers and probe from Callahan et al. [17] targeting the 3D region of the viral genome. All virus/cell combinations where at least two of three replicate cultures were PCR-positive were then titrated on adherent BHK-21 cells as previously described [12].

### 2.4. Growth Curves 

The growth kinetics of Asia-#9, Asia-#8, Asia-Mut 9-4, Asia-Mut 9-7, Asia-Mut 9-9 and Asia-Mut 8-8 were determined in BHK-2P cells, seeded with a density of 1 × 10^6^ cells/mL and infected at a multiplicity of infection of 0.01. Cells were incubated for 24 h, at 37 °C, 80% humidity, 5% CO_2_ and 320 rpm and supernatants were collected at 0, 4, 8, 12, 16, 20 and 24 h after inoculation. Virus titers (TCID_50_/mL) were determined as described above. 

### 2.5. Vaccine Formulation

Adherent BHK-21 cells were infected with Asia-1 Shamir and BHK-2P suspension cells were infected with Asia-#8, Asia-#9, Asia-Mut 9-4 and Asia-Mut 9-7. Cell culture supernatant was harvested after separation of the cell debris by centrifugation. The supernatant was inactivated with 0.1 M binary ethyleneimine (BEI) (Sigma-Aldrich) for ≥24 h at 30 °C, concentrated with 8% (*w*/*v*) polyethylene glycol 6000 (Sigma-Aldrich), clarified by centrifugation and resuspended in 5–10% of the initial volume in Tris buffer (0.02 M Tris, 0.25 M sodium chloride, pH 7.5). Sarcosine solution (Sigma-Aldrich) was added in a ratio of 4:1 and the mixture was stored at 4 °C for 30 min before loading on 15–45% (*w*/*v*) sucrose density gradients (SDG) as previously described [18]. Virus particles were resolved by centrifugation at 16,400× *g* for 16 h at 4 °C. The gradients were collected in 1 mL fractions and were analyzed spectrophotometrically by measuring the optical density (OD) at 260 nm. This procedure was also used to assess the particle stability.

For vaccine preparation, the two to four fractions with the highest OD were then pooled, diluted with NET buffer (0.05 M Tris, 0.1 M sodium chloride, 0.004 M sodium-EDTA, pH 8.0) at a proportion of virus/NET buffer of 1:3 and were pelleted at 32,000× *g* for 16 h at 4 °C. The pellet was resuspended in 200 µL NET buffer. The content of 146S particles (i.e., intact FMD virions) was calculated using the extinction coefficient E_260nm_ = 131.6 [19]. Vaccines were formulated as water-in-oil-in-water (W/O/W) emulsions with Montanide ISA 206 VG adjuvant (Seppic, Paris, France), each preparation containing 4 µg/mL of BEI-inactivated, SDG-purified FMDV antigen. The adjuvant was mixed into the aqueous antigen phase (54/46 *v*/*v*) at 35 °C for 5 min. The formulation was rested at room temperature (RT) for 30 min and then stored at 4 °C for no longer than 24 h before application.

### 2.6. Guinea Pig Immunization

Hartley guinea pigs of either sex weighing about 300–350 g (Charles River Laboratories) were divided into six groups of two animals each. Immunization of guinea pigs was performed by intramuscular (IM) injection of 0.5 mL of the vaccine formulation described above. Group 1 received a commercially available inactivated quadrivalent FMDV vaccine (positive control, PC). Group 2 received the Asia-1 Shamir vaccine formulation as control for the vaccine purification and formulation process and groups 3 to 6 received Asia-#8, Asia-#9, Asia-Mut 9-4 and Asia-Mut 9-7, respectively. All animals were bled on day 0 and day 21 post-vaccination (dpv). 

### 2.7. Ethics Statement

All work with living animals occurred after ethical review and in compliance with local, state, and national animal welfare regulations. The experimental protocol was filed with the State Office for Agriculture, Food and Fisheries of Mecklenburg-Vorpommern, the competent authority for animal experiments conducted at the Insel Riems site of the FLI (file no. 7221.3-2-042/17). The animals were handled in accordance with the applicable European and German guidelines for the use of experimental animals by researchers certified by the Federation of European Laboratory Animals Science Associations.

### 2.8. Virus Neutralization (VN) Test 

Neutralizing antibodies against FMDV Asia-1 Shamir in serum samples collected at 0 and 21 dpv from guinea pigs were measured as prescribed by the World Organisation for Animal Health (OIE) [20] using adherent BHK-21 cells. The antibody titers were calculated as the log_10_ of the reciprocal of the final serum dilution where 50% of wells are protected. The antigenic match between vaccine candidates and the original Asia-1 Shamir isolate is expressed as the ratio (r_1_) between the titer of the serum against the original isolate and the titer of the serum against the homologous vaccine virus [20].

### 2.9. Temperature and pH Sensitivity

Storage conditions at different temperatures and sensitivity towards decreasing pH were examined to test whether the acquired mutations of Asia-#8 and Asia-#9 led to a reduced stability of the virus particle compared to the original Asia-1 Shamir isolate. For pH testing, a protocol by Martín-Acebes et al. [21] was used with previously described modifications [12]. Solutions of pH 8.0, 7.5, 7.0, 6.5, 6.0 and 5.5 were tested with an incubation time of 30 min. 

To examine the stability of the virus particle at the different storage conditions encountered during the vaccine formulation process, equal amounts of virus (Asia-1 Shamir, Asia-#8 or Asia-#9) were added in a final dilution of 1:100 to 1.5 mL of serum-free Cellvento™ BHK200 media (Merck). The samples were stored at 4 °C, room temperature (RT) or 37 °C with agitation (350 rpm) for 8, 24, or 48 h. The remaining infectivity was determined as described above by estimation of viral titers by endpoint titration. All experiments were independently performed three times.

### 2.10. Statistical Analysis 

The differences between treatment groups were evaluated using one-way ANOVA, combined with Tukey’s multiple comparisons test or rather *t*-tests to examine differences between two data sets in GraphPad Prism 8 (http://www.graphpad.com). *p*-values of <0.001 were considered significant.

## 3. Results

### 3.1. Generation of Recombinant FMD Viruses with Mutations Observed in Passage-Derived Strains

In order to determine the minimum of adaptation necessary for efficient replication of FMDV Asia-1 in BHK-2P suspension cells, a panel of virus mutants was constructed based on the amino acid exchanges seen in the previously described passage-derived strains Asia-#8 and Asia-#9 [12]. Asia-#8 has two amino acid exchanges on the capsid surface of VP1 (Q108R and Q110R), while Asia-#9 has three amino acid exchanges in VP1 (T83A, Q110K and E202K) and one exchange in VP3 (E59K). One to four point mutations, alone or in combination, were introduced in the full-length cDNA clone of FMDV serotype Asia-1 Shamir (pT7S3_Asia-1_GTG) and the in-vitro synthesized RNA transcripts were transfected into adherent BHK-21 monolayers. The adherent BHK cell line is susceptible to all FMDV strains used in this study. In total, 17 different viable recombinant viruses were rescued (Table 2). Sequencing of the entire capsid-coding region of the rescued virus mutants after two passages in adherent BHK-21 cells confirmed that they were identical to the parental virus apart from the desired amino acid substitutions in the VP1 and/or VP3 protein. 

### 3.2. Characterization of the Recovered FMDV Mutants and Their Parental Strains

The constructed virus mutants, as well as the original Asia-1 Shamir isolate and its passaged derivatives Asia-#8 and Asia-#9 [12] were cultured on a set of different cell lines to investigate their receptor usage and find the mutants that grow in BHK-2P suspension cells. Adherent BHK-21 cells served as a positive control. CHO-K1 cells were used because they present heparan sulfate proteoglycans (HSPG) but no integrins on the cell surface, while CHO677 cells do not present either and are resistant to “wild type” FMD viruses. Apart from the BHK-2P suspension cell line, two more suspension BHK cell lines were tested: BHK-InVitrus (here referred to as cell line #8), the “mother cell line” of FMDV strain Asia-#8, and production BHK (#9), the “mother cell line” of virus strain Asia-#9 [12]. The cultures were harvested 24h post infection and FMDV RNA content was quantified by amplification of the 3D region of the viral FMDV genome using RT-qPCR. All virus/cell combinations for which at least two of three replicate cultures were PCR-positive were titrated on adherent BHK-21 cells. RT-qPCR results and titers were generally well correlated (see Table S1 and Figure S1 in the Supplementary Material). 

The passage-derived Asia-#8 strain is characterized by two amino acid exchanges in VP1, Q108R and Q110R, which allow for this strain to infect and replicate in all tested cell lines. Three virus mutants were constructed with either one or both of these exchanges. The recombinant Asia-Mut 8-3, which has the same capsid mutations as the original Asia-#8 strain, grew to similar titers on all cell lines except CHO-K1, where its titer was about 200-fold lower than in adherent BHK-21 cells. Unlike the passage-derived Asia-#8 strain, mutant 8-3 did not grow on CHO677 cells. 

The exchange of only one of the amino acids, either Q108R or Q110R, did not enable infection of and/or replication in BHK-2P suspension cells or any of the adherent CHO cell lines, but the mutants 8-1 and 8-2 grew to titers of >6 log_10_ TCID_50_/mL in the BHK-InVitrus suspension cell line (#8) and to reduced titers in the production BHK suspension cell line (#9) (Table 3). 

The Asia-#9 strain has four amino acid exchanges in the viral capsid: T83A, Q110K and E202K in VP1 and E59K in VP3. The majority of the 14 recombinant viruses that were constructed based on the amino acid exchanges seen in Asia-#9 did not grow in BHK-2P suspension cells. The original Asia-1 Shamir isolate, all mutants with only one of the four mutations and almost all double mutants were only able to infect and replicate in BHK-InVitrus (#8) and to a lesser extent, in production BHK (#9) suspension cells (Table 4). 

The quadruple mutant Asia-Mut 9-9 was able to infect and replicate in all tested cell lines and grow to titers similar to the passage-derived Asia-#9 strain. Apart from this mutant, four other recombinant viruses did grow in BHK-2P cells: Asia-Mut 9-4, Asia-Mut 9-7, Asia-Mut 9-10 and Asia-Mut 9-12. All mutants share the E202K amino acid exchange, in addition to either E59K or Q110K alone or in combination with T83A. The combination of E202K and T83A alone (as in Asia-Mut 9-13) does not allow infection of and/or replication in BHK-2P suspension cells. 

Notably, the ability to infect and replicate in BHK-2P suspension cells was not linked to the ability to infect and replicate in CHO677 and/or CHO-K1 cells. While Asia-Mut 9-4, Asia-Mut 9-10 and Asia-Mut 9-12 replicated in all tested suspension BHK cell lines, but not in the CHO cells, the inclusion of the amino acid exchanges T83A together with Q110K and E202K in Asia-Mut 9-7, conferred the ability to also infect and replicate in CHO-K1 and CHO677 cells. The combination of E202K and Q110K (with or without T83A) was advantageous for growth in BHK-2P cells compared to the combination of E202K and E59K (with or without T83A). While Asia-Mut 9-4 and Asia-Mut 9-7 developed titers similar to the original Asia-#9 isolate, Asia-Mut 9-10 and Asia-Mut 9-12 grew to lower titers in BHK-2P cells (Table 5). 

### 3.3. Recombinant FMDV Variants Have Altered Growth Properties Compared to Passage-Derived Strains 

In order to determine the best harvest time-point for vaccine antigen production and to detect differences in replication efficiency in BHK-2P cells, the growth kinetics of the recombinant virus Asia-Mut 8-3 were compared to the passage-derived strain Asia-#8. Similarly, the recombinant viruses Asia-Mut 9-4, Asia-Mut 9-7 and Asia-Mut 9-9 were compared to the passage-derived strain Asia-#9. Mutants 9-10 and 9-12 were not tested further since they had grown to considerably lower titers in BHK-2P cells than the Asia-#9 strain in the previous experiment. 

**Figure 1:**
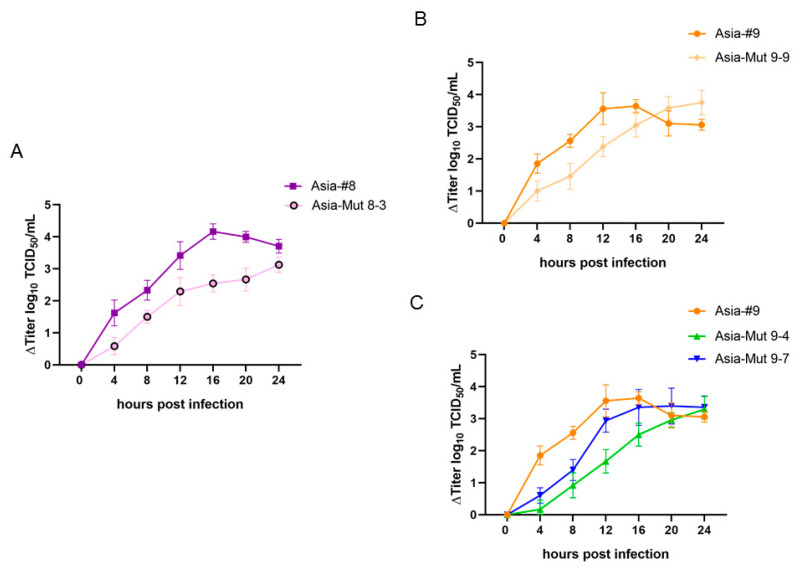
Growth curves in BHK-2P cells of the recombinant viruses, Asia-Mut 8-3 (**A**), Asia-Mut 9-9 (**B**), Asia-Mut 9-4 and Asia-Mut 9-7 (**C**) in comparison to the corresponding passage-derived strains Asia-#8 and Asia-#9. All values are plotted relative to the titer at 0 hours to emphasize the change in virus titer over time.

The growth properties of the recombinant viruses differed from the original passage-derived strains. The titers of both Asia-#8 and Asia-#9 peaked at 16 hpi (Asia-#8: 8.0 ± 0.2 log_10_ TCID_50_/mL, Asia-#9: 7.6 ± 0.2 log_10_ TCID_50_/mL), followed by a decrease in viral titer until 24 hpi, while the recombinant viruses grew slower and their titers steadily increased until the end of the experiment at 24 hpi (Figure 1). 

In more detail, the recombinant virus Asia-Mut 8-3, which contains the same amino acid exchanges in the viral capsid as passage-derived Asia-#8, had significantly lower titers than Asia-#8 at all time points (Figure 1A). Asia-Mut 9-9, which contains the same amino acid exchanges in the viral capsid as Asia-#9, grew more slowly than Asia-#9, too, with significantly lower titers until 16 hpi. However, the final titer at 24 hpi (7.8 ± 0.3 log_10_ TCID_50_/mL) was similar to the peak titer of Asia-#9 at 16 hpi (Figure 1B). Asia-Mut 9-4, containing the substitutions Q110K and E202K in VP1, replicated the slowest with a consistent increase in viral titer over the whole 24 h, reaching a final titer at 24 hpi of 7.0 ± 0.6 log_10_ TCID_50_/mL, similar to Asia-#9 at the same time point (7.0 ± 0.2 log_10_ TCID_50_/mL). For Asia-Mut 9-7, which has all three Asia-#9 mutations in VP1 (T83A, Q110K and E202K), the peak titer was reached at 24 hpi (7.3 ± 0.2 log_10_ TCID_50_/mL) and was similar to the value of Asia-#9 at 24 h although its growth kinetics showed a strong increase in titer until 16 hpi followed by a plateau (Figure 1C). Furthermore, it is worth mentioning that while Asia-Mut 9-7 had efficiently lysed the majority of cells after 24 hpi, cells infected with Asia-Mut 9-4 remained at a high viability of 80-90% during the entire experiment (data not shown).

### 3.4. Immunogenicity of Passage-Derived Strains and Recombinant Viruses

The passage-derived strains Asia-#8 and Asia-#9 were chosen for vaccine formulation over the recombinant viruses Asia-Mut 8-3 and Asia-Mut 9-9 because of their better growth in BHK-2P suspension cells. Similarly, Asia-Mut 9-10 and Asia-Mut 9-12 were dismissed because of their low titers in these cells. Four isolates were used as vaccine candidates: Asia-#8, Asia-#9, Asia-Mut 9-4 and Asia-Mut 9-7. Additionally, a vaccine was formulated from the original Asia-1 Shamir isolate to serve as process control and a commercially available vaccine based on the same isolate was used as positive control (PC). The titers of the virus preparations before inactivation and formulation were 10^7.9^ TCID_50_/mL for the original Asia-1 Shamir isolate, 10^7.5^ for Asia-#8, 10^7.7^ for Asia-#9, 10^7.7^ for Asia-Mut 9-4 and 10^7.3^ for Asia-Mut 9-7. 

Two guinea pigs were immunized with each vaccine preparation, blood was collected 21 days after vaccination and antibodies were quantified in a virus neutralization (VN) test. Both Asia-1 Shamir vaccines (commercial and in-house) induced neutralizing antibodies with equivalent titers (1.8 ± 0.16 log_10_ and 1.9 ± 0.27 log_10_, respectively) (Table 6). The vaccine candidates formulated from Asia-#8 and Asia-#9 did not induce any detectable neutralizing antibodies, neither against the heterologous Asia-1 Shamir “wild type” isolate, nor against the homologous passage-derived strains used for vaccine preparation. Sera from guinea pigs immunized with Asia-Mut 9-4 did not contain any detectable neutralizing antibodies against the original Asia-1 Shamir isolate but had a neutralizing titer of 0.94 ± 0.05 log_10_ against the homologous Asia-Mut 9-4 recombinant virus. Only the vaccine candidate Asia-Mut 9-7 induced detectable neutralizing antibodies (0.75 log_10_) against the heterologous Asia-1 Shamir strain with an r_1_-value (neutralizing titer ratio) of 0.4 relative to the PC. 

### 3.5. Virus Strains Asia-#8 and Asia-#9 Do Not Differ in pH and Temperature Sensitivity from the Parental Isolate Asia-1 Shamir but their Virus Particles Are Less Stable 

**Figure 2:**
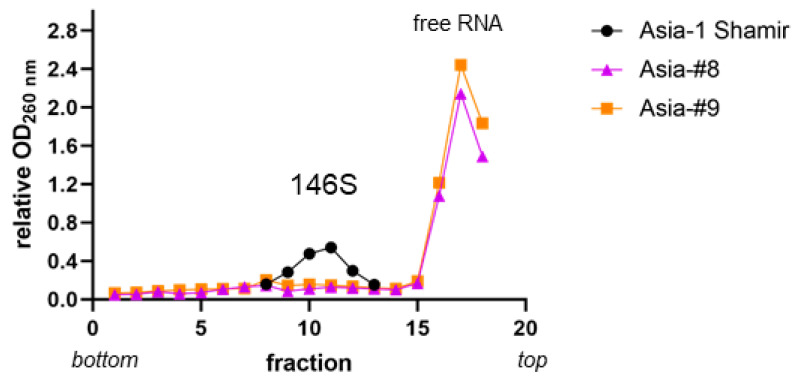
Sucrose gradient profiles of Asia-1 Shamir, Asia-#8 and Asia-#9. Virus strains Asia-#8 and Asia-#9 were grown in BHK-2P and the wild-type isolate Asia-1 Shamir was grown in adherent BHK-21 cells. The harvested virus was concentrated by ultracentrifugation and sedimented through a 15-45% sucrose density gradient. The peaks corresponding to RNA in 146S particles and free RNA are indicated.

Because the vaccines formulated from Asia-#8 and Asia-#9 were not immunogenic, virus preparations were examined on sucrose density gradients to compare the yield of intact FMD virions (146S particles). Spectrophotometric measurements revealed a lower 146S peak and high amounts of free RNA for Asia-#8 and Asia-#9 in comparison to Asia-1 Shamir (Figure 2). 

**Figure 3:**
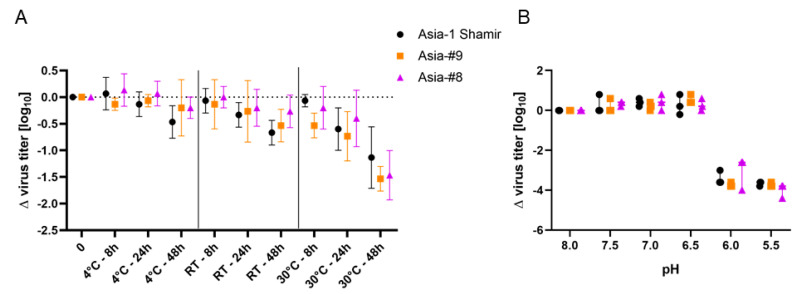
Temperature and pH stability of Asia-1 Shamir, Asia-#8 and Asia-#9. Viruses were kept at neutral pH at 4 °C, RT and 37 °C for 8, 24 and 48 h (**A**) as well as at room temperature in buffered solutions at pH 8.0, 7.5, 7.0, 6.5, 6.0 and 5.5 for 30 min (**B**). Values on the y-axis represent the reduction in titer compared to time point zero (**A**) or to the virus incubated at pH 8.0 (**B**). All experiments were independently performed three times.

The virus stability at different temperatures and time points, as well as the pH-dependent inactivation of Asia-#8 and Asia-#9 was compared to the parental isolate Asia-1 Shamir. Viral titers of all viruses declined over time at all tested temperatures. However, while the loss in titer at 4 °C and RT was not significant compared to the initial titer, the titer of all viruses significantly dropped after 48 h at 30 °C. Statistical analysis did not reveal any significant differences between the three tested viruses at any temperature and time point (Figure 3A), and the pH-dependent inactivation kinetics of Asia-#8, Asia-#9 and Asia-1 Shamir were similar as well (Figure 3B).

## 4. Discussion

BHK-21 cells are the most common cell line for the production of FMDV vaccine antigen, but the adaptation from adherence to growth in suspension can cause profound changes in the cells that the virus in turn needs to adapt to [22]. It is known that BHK cells change on repeated subculturing and can even lose their susceptibility for FMDV [10], with serotype Asia-1 viruses reportedly being most affected by this exclusion [9]. Previous investigations showed that the resistance of a certain BHK cell line can be mitigated by using a closely related but permissive cell line as a “wet nurse” for the adaption of the virus [12]. However, viral adaptation caused several capsid mutations that cast doubt on the suitability of these highly adapted strains as vaccine candidates. In this context, the targeted insertion of a minimal set of necessary capsid mutations to overcome FMDV resistance in the production cell line of choice would be of high importance for the efficient production of FMDV vaccine antigen. 

The construction of a panel of recombinant viruses precipitated several interesting findings: The E202K amino acid exchange seems to be of pivotal importance to overcome the resistance of BHK-2P cells to FMDV serotype Asia-1. This residue is located on the edge of the protomer at the interface of VP1 and VP3 [23]. In combination with at least one other amino acid exchange that adds a positive charge on the outer surface of the particle (Q110K in VP1 or E59K in VP3), the E202K substitution allows FMDV Asia-1 to infect BHK-2P cells. Of these two pairings, the two exchanges in VP1 lead to significantly (*p* < 0.0001) higher virus titers. 

Because the passage-derived strains Asia-#8 and Asia-#9 were both able to infect CHO677 cells in addition to BHK-2P, we previously hypothesized that infection of BHK-2P cells is only possible for viruses that do not require integrins or heparan sulfate proteoglycans (HSPG) to attach to the cell surface [12]. However, the observation that the recombinant viruses Asia-Mut 9-4, Asia-Mut 9-10 and Asia-Mut 9-12 were able to infect BHK-2P, but not CHO-K1 and CHO677 cells, now disproved this hypothesis. Therefore, it remains unknown what receptor (or receptors) FMDV uses to attach to BHK-2P cells, but the acquisition of positive charges on the viral capsid seems to be advantageous. It would be helpful to perform plaque assays in the adherent cell lines to see if there are differences in plaque phenotypes. In addition, the specific infectivity, i.e., the ratio of virus particles to infectious units, should be determined for the Asia-1 Shamir wild-type isolate, as well as for the passage-derived strains and the recombinant viruses. It is possible that mutations in the capsid reduce the specific infectivity compared to the wild-type isolate. 

Residue 110 in VP1 is located next to the five-fold symmetry axis of the virus capsid pentamer [12]. With the amino acid exchange T83A in addition to Q110K (and E202K) in Asia-Mut 9-7, a second amino acid exchange next to the five-fold symmetry axis took place. This mutant was then able to infect CHO-K1 and CHO677 cells, which supports the assumption that amino acid exchanges in this region are associated with the binding of a third, non-integrin and non-HSPG FMDV receptor [24,25], which may be the Jumonji C-domain containing protein 6 [26]. As evidenced by Asia-Mut 9-4, the third and fourth mutation found in Asia-#9 (in addition to E202K and Q110K) were not strictly required to infect BHK-2P cells. However, it seems to be of advantage for the virus to acquire additional mutations around the five-fold symmetry axis to better exploit this unknown receptor as demonstrated by the higher titers attained by Asia-Mut 9-7. On the other hand, the single mutants based on the Asia-#8 strain revealed that the acquisition of both its amino acid exchanges (Q108R and Q110R) around the five-fold symmetry axis was essential in the absence of other adaptive mutations. By itself, neither mutation was sufficient to infect BHK-2P cells. Because both Asia-#8 and Asia-#9 make use of mutations around the five-fold symmetry axis, it can be concluded that the unknown receptor is ubiquitous on the surface of all BHK and CHO cells tested in this study and is not restricted to a certain cell line. 

Interestingly, there are two mutants (Asia-Mut 9-10 and Asia-Mut 9-12), which cannot use HSPG receptors (since they cannot infect CHO-K1 cells), do not have any amino acid exchanges next to the five-fold axis, but nevertheless can infect BHK-2P cells. This may be an indication of yet another FMDV receptor that remains to be identified.

The susceptibility of the three BHK suspension cell lines also varied. Infection of BHK-2P cells was only possible for the few mutants discussed above, whereas all recombinant viruses were able to infect BHK-InVitrus and replicate to moderately high titers. Finally, infection of production BHK was possible for all mutants except Asia-Mut 9-3, but high titers in these cells were only attained by the mutants that could also infect BHK-2P. This might indicate either a different set of available receptors between the suspension cell lines or the expression of the same receptor in different quantities. 

The growth curves revealed a different progression of virus replication for Asia-Mut 9-4 and Asia-Mut 9-7 compared to the parental Asia-#9 strain. Similarly, both Asia-Mut 9-9 and Asia-Mut 8-3 were different from the respective passage-derived strains Asia-#9 and Asia-#8, despite having the same amino acid exchanges in the viral capsid proteins. All recombinant viruses replicated significantly slower in the BHK-2P cells than their parental strains. In addition to the alterations of the viral capsid, Asia-#8 and Asia-#9 have an amino acid exchange (K285Q) in the non-structural 2C protein [12], which was not introduced into the recombinant viruses. The highly conserved 2C protein is a membrane-binding component of the virus replication complex, responsible for RNA replication and the induction of apoptosis in BHK cells among other functions [27,28,29]. The 2C protein also interacts with cellular vimentin which builds cage-like structures around the 2C protein during FMDV infection [30] but whose biosynthesis is decreased in suspension cells [31]. The vimentin cages of different BHK cell lines may differ in their interaction with the FMDV 2C protein, and we had previously hypothesized that the mutation in the 2C gene enhances viral replication [12] in the context of the altered vimentin synthesis in BHK suspension cells [31]. The slower replication of the recombinant strains lacking this mutation strengthens this hypothesis, especially because the recombinant Asia-Mut 9-4 was not able to efficiently lyse BHK-2P cells after infection. The missing mutation in the 2C gene might be the reason for the adverse effect on virus replication in the mutants compared to the passage-derived strains with the same capsid protein sequence. The growth kinetics of Asia-Mut 9-10 and Asia-Mut 9-12 were not assessed because these viruses had already exhibited decreased viral titers on BHK-2P in the infectivity testing.

The preparation of in-house vaccines and the immunization of guinea pigs revealed a presumably protective heterologous antibody response with an r_1_-value of 0.4 only for the Asia-Mut 9-7 recombinant. Generally speaking, an r_1_ > 0.3 suggests that there is a sufficiently close relationship between field isolate and vaccine virus to allow adequate cross-protection, an r_1_ < 0.3 indicates that the field isolate is so different from the vaccine that the vaccine is unlikely to protect [20]. 

Immunization with Asia-Mut 9-4 only induced neutralizing antibodies against the homologous Asia-Mut 9-4 virus but not against Asia-1 Shamir. This supports the conclusion that this preparation successfully induced the production of specific antibodies in the guinea pigs, but the amino acid exchanges in the Asia-Mut 9-4 virus led to insufficient neutralization of the original isolate. This finding is quite surprising for several reasons. Asia-Mut 9-4 contains only two amino acid exchanges (Q110K, E202K), unlike Asia-Mut 9-7 that has an additional amino acid exchange (T83A) in VP1. None of the introduced amino acid exchanges are part of any described antigenic site of Asia-1 [32,33,34,35,36,37]. The C-terminus of VP1 has been discussed to be part of the major antigenic site 1 located within the surface-exposed G-H loop [33], but other studies could not confirm the described antigenicity in the context of serotype Asia-1 [32]. In addition, the residue 202 is located at the interface of VP1 and VP3 and is not exposed on the external surface of the virion [23].

Unexpectedly, vaccines made with the adapted virus strains Asia-#8 and Asia-#9 did not induce the production of neutralizing antibodies at all. The examination of the virus particle integrity revealed only low amounts of intact 146S particles in comparison to the original Asia-1 Shamir isolate, but in addition to the spectrophotometric quantification of RNA in the gradient fractions, the gradient fractions should be tested for FMDV capsid protein by Western blot or antigen ELISA.

An analysis of secondary RNA structures could provide more insight into possible reasons for the observed low amount of intact viral particles. The mutations in the capsid-coding regions of the adapted strains could interfere with the encapsidation of the viral RNA and the formation of mature virions, but the observed good correlation between viral genome replication (Ct value in the RT-qPCR) and intact particle yield (virus titer) does not indicate such an impairment. 

FMDV is naturally highly sensitive to even mildly acidic pH [38] and the maintenance of the cold chain is of high importance for FMDV vaccines [39]. It was therefore examined if the adapted strains are more sensitive against changes in pH or temperature than the original Asia-1 Shamir isolate, but we found no difference in the impact of pH or temperature on virus particle instability for the adapted virus strains.

## 5. Conclusions

This study successfully demonstrated the utility of genetic engineering for FMDV vaccine production. Through the targeted insertion of a minimum set of known adaptive mutations in the viral genome, it is possible to immediately enable viral growth in BHK-2P suspension cells without the inconvenience of a time-consuming step-wise adaptation of the virus in cell culture.

## Figures and Tables

**Table 1:** Cell lines used in the study.

Cell Line	Alternative Names	Growth	Collection	Accession Number
BHK-21	BHK-21 clone “Tübingen”	adherent	CCLV ^1^	RIE 164
BHK-2P	BHK21CL13-2P	in suspension	ECACC ^2^	84111301
BHK-InVitrus	“cell line #8”	in suspension	ECACC	05062302
production BHK	“cell line #9”	in suspension	Merck KGaA	n/a
CHO-K1		adherent	ATCC ^3^	CCL-61
CHO677	PgsD-677	adherent	ATCC	CRL 2244

^1^ Collection of Cell Lines in Veterinary Medicine, FLI, Greifswald, Germany. ^2^ European Collection of Authenticated Cell Cultures, Public Health England, Porton Down, United Kingdom. ^3^ American Type Culture Collection, Manassas, USA.

**Table 2:** Recombinant FMD virus mutants and their corresponding mutations as well as the primer and plasmid backbone used for their construction.

Construct	Introduced Mutation/ Amino Acid Exchange	Primers	Backbone
pT7S3_Asia-1	GTT to GTG in 2C gene	FMD-5143-F ^a^	pT7S3_Asia-1
		Asia 2C_GTT-GTG-Rev	
Asia-Mut 8-1	Q108R	Asia#8_VP1_Q108R-Fw	pT7S3_Asia-1_GTG
		FMD-4303-R ^a^	
Asia-Mut 8-2	Q110R	Asia#8_VP1_Q110R-Fw	pT7S3_Asia-1_GTG
		FMD-4303-R ^a^	
Asia-Mut 8-3	Q108R + Q110R	Asia#8_Q108R_Q110R-Fw	pT7S3_Asia-1_GTG
		FMD-4303-R ^a^	
Asia-Mut 9-1	E59K	Asia#9_VP3_E59K-Fw	pT7S3_Asia-1_GTG
		FMD-3223-R ^a^	
Asia-Mut 9-2	E59K + T83A	Asia#9_VP3_E59K-Fw	pT7S3_Asia-1_GTG
		Asia#9_VP1_T83A-Rev	
Asia-Mut 9-3	T83A	FMD-3161-F ^a^	pT7S3_Asia-1_GTG
		Asia#9_VP1_T83A-Rev	
Asia-Mut 9-4	Q110K + E202K	Asia#9_VP1_Q110K-Fw	pT7S3_Asia-1_GTG
		Asia#9_VP1_E202K-Rev	
Asia-Mut 9-5	Q110K	Asia#9_VP1_Q110K-Fw	pT7S3_Asia-1_GTG
		FMD-4303-R ^a^	
Asia-Mut 9-6	E202K	FMD-3161-F ^a^	pT7S3_Asia-1_GTG
		Asia#9_VP1_E202K-Rev	
Asia-Mut 9-7	T83A + Q110K + E202K	FMD-3161-F ^a^	pT7S3_Asia-1_Mut 4
		Asia#9_VP1_T83A-Rev	
Asia-Mut 9-8	E59K + T83A + Q110K	Asia#9_VP1_Q110K-Fw	pT7S3_Asia-1_Mut 2
		FMD-4303-R ^a^	
Asia-Mut 9-9	E59K + T83A + Q110K + E202K	Asia#9_VP3_E59K-Fw	pT7S3_Asia-1_Mut 4
		Asia#9_VP1_T83A-Rev	
Asia-Mut 9-10	E59K + T83A + E202K	FMD-3161-F ^a^	pT7S3_Asia-1_Mut 2
		Asia#9_VP1_E202K-Rev	
Asia-Mut 9-11	T83A + Q110K	megaprimer of mutant 3	pT7S3_Asia-1_Mut 5
Asia-Mut 9-12	E59K + E202K	megaprimer of mutant 1	pT7S3_Asia-1_Mut 6
Asia-Mut 9-13	T83A + E202K	megaprimer of mutant 3	pT7S3_Asia-1_Mut 6
Asia-Mut 9-14	E59K + Q110K	megaprimer of mutant 1	pT7S3_Asia-1_Mut 5

^a^ from Dill et al. [14].

**Table 3:** Mean and standard deviation of viral titers (log_10_ TCID_50_/mL) of virus strain Asia-#8 and the corresponding recombinant viruses.

Isolate	Amino Acid Exchange	adh. BHK-21	CHO-K1	CHO677	BHK-2P	BHK- InV (#8)	Prod. BHK (#9)
Asia-#8	Q108R+Q110R	8.3 ± 0.3	6.0 ± 0.2	4.7 ± 0.4	7.8 ± 0.3	7.7 ± 0.3	8.3 ± 0.4
Asia-Mut 8-1	Q108R	7.6 ± 0.1	n.t.	n.t.	n.t.	6.6 ± 0.3	4.6 ± 0.3
Asia-Mut 8-2	Q110R	7.7 ± 0.3	n.t.	n.t.	n.t.	6.8 ± 0.5	5.8 ± 0.3
Asia-Mut 8-3	Q108R+Q110R	7.7 ± 0.2	6.0 ± 0.2	n.t.	7.2 ± 0.3	7.4 ± 0.2	7.6 ± 0.1

n.t.: screened out by PCR, not titrated.

**Table 4:** Mean and standard deviation of viral titers (log_10_ TCID_50_/mL) of recombinant viruses based on Asia-#9 that were not able to infect and replicate in BHK-2P suspension cells. A lack of replication was assumed for all virus/cell combinations for which fewer than two of three replicate cultures were positive in the FMDV RT-qPCR.

Isolate	Amino Acid Exchange	adh. BHK-21	CHO-K1	CHO677	BHK-2P	BHK InV (#8)	Prod. BHK (#9)
Asia-1 Shamir	none	7.7 ± 0.3	n.t.	n.t.	n.t.	6.3 ± 0.3	3.0 ± 0.3
Asia-Mut 9-1	E59K	7.8 ± 0.2	n.t.	n.t.	n.t.	6.8 ± 0.4	4.7 ± 0.3
Asia-Mut 9-2	E59K+T83A	7.5 ± 0.2	n.t.	n.t.	n.t.	6.9 ± 0.3	4.8 ± 0.2
Asia-Mut 9-3	T83A	7.7 ± 0.3	n.t.	n.t.	n.t.	5.5 ± 0.3	n.t.
Asia-Mut 9-5	Q110K	7.8 ± 0.4	n.t.	n.t.	n.t.	6.9 ± 0.3	4.9 ± 0.3
Asia-Mut 9-6	E202K	7.7 ± 0.2	n.t.	n.t.	n.t.	6.5 ± 0.1	4.1 ± 0.3
Asia-Mut 9-8	E59K+T83A+ Q110K	7.9 ± 0.4	n.t.	n.t.	n.t.	6.3 ± 0.1	5.4 ± 0.3
Asia-Mut 9-11	T83A+Q110K	7.6 ± 0.2	n.t.	n.t.	n.t.	6.1 ± 0.3	5.8 ± 0.2
Asia-Mut 9-13	T83A+E202K	7.3 ± 0.3	n.t.	n.t.	n.t.	6.6 ± 0.3	5.3 ± 0.3
Asia-Mut 9-14	E59K+Q110K	7.7 ± 0.4	n.t.	n.t.	n.t.	6.8 ± 0.2	6.2 ± 0.5

n.t.: screened out by PCR, not titrated.

**Table 5:** Mean and standard deviation of viral titers (log_10_ TCID_50_/mL) of virus isolate Asia-#9 and the corresponding recombinant viruses that can infect and replicate in BHK-2P cells. Replication was assumed for all virus/cell combinations for which at least two of three replicate cultures were positive in the FMDV RT-qPCR.

Isolate	Amino Acid Exchange	Adh. BHK-21	CHO-K1	CHO677	BHK-2P	BHK- InV (#8)	Prod. BHK (#9)
Asia-#9	E59K + T83A + Q110K+E202K	7.5 ± 0.3	6.0 ± 0.6	5.8 ± 0.2	6.8 ± 0.3	6.8 ± 0.2	7.7 ± 0.3
Asia-Mut 9-4	Q110K + E202K	7.6 ± 0.2	n.t.	n.t.	7.7 ± 0.4	7.5 ± 0.5	8.0 ± 0.2
Asia-Mut 9-7	T83A + Q110K + E202K	7.4 ± 0.1	5.8 ± 0.2	6.0 ± 0.3	6.8 ± 0.3	6.9 ± 0.3	7.7 ± 0.3
Asia-Mut 9-9	E59K + T83A + Q110K + E202K	8.1 ± 0.3	5.3 ± 0.4	5.5 ± 0.3	7.2 ± 0.4	7.4 ± 0.2	7.7 ± 0.4
Asia-Mut 9-10	E59K + T83A+ E202K	7.6 ± 0.3	n.t.	n.t.	4.8 ± 0.2	7.3 ± 0.5	7.5 ± 0.2
Asia-Mut 9-12	E59K + E202K	7.7 ± 0.3	n.t.	n.t.	5.8 ± 0.4	7.3 ± 0.5	7.7 ± 0.3

n.t.: screened out by PCR, not titrated.

**Table 6:** Virus neutralization titers for the vaccine candidates and the corresponding r_1_-values relative to the PC.

Isolate	Serum Titer [log_10_]		r_1_-Value
	Heterologous	Homologous	
PC	-	1.8 ± 0.16	1
Asia-1 Shamir	-	1.9 ± 0.27	1.1
Asia-#8	0	0	0
Asia-#9	0	0	0
Asia-Mut 9-4	0	0.94 ± 0.05	0
Asia-Mut 9-7	0.75 ± 0.00	1.28 ± 0.00	0.4

## Supplementary Materials

The following are available online at https://www.mdpi.com/2076-393X/8/4/583/s1, Figure S1: Correlation between FMDV RT-qPCR and virus titration. Table S1: FMDV RT-qPCR results for the infectivity assay.
